# Overexpression of apelin in Wharton’ jelly mesenchymal stem cell reverses insulin resistance and promotes pancreatic β cell proliferation in type 2 diabetic rats

**DOI:** 10.1186/s13287-018-1084-x

**Published:** 2018-12-07

**Authors:** Lian Ru Gao, Ning Kun Zhang, Yan Zhang, Yu Chen, Li Wang, Ying Zhu, Hai Hong Tang

**Affiliations:** 1 Center of Cardiology, The Sixth Medical Center of P.L.A. General Hospital (Former Navy General Hospital), NO.6 Fucheng Road, Beijing, Haidian District 100048 People’s Republic of China; 2 Department of Internal Medicine, The 413th Hospital of P.L.A. 98 Wenhua Road Zhoushan, Zhejiang, 316000 People’s Republic of China

**Keywords:** Apelin, Type 2 diabetes, Wharton’s jelly, Mesenchymal stem cells, Lentiviral transduction, ß cell proliferation

## Abstract

**Background:**

Apelin plays a key beneficial role in energy metabolism by increasing glucose uptake and insulin sensitivity; however, apelin has a short half-life because it is rapidly cleared from the circulation limiting its therapeutic benefit. The aim of this study is to create a new approach to treat type 2 diabetes by inducing prolonged expression of apelin in Wharton’s jelly-derived mesenchymal stem cells (WJ-MSCs).

**Methods:**

A type 2 diabetic rat model was given a high-fat diet combined with low-dose streptozotocin (STZ) injection. The human WJ-MSCs were isolated and subsequently transduced with apelin-expressing lentiviral particles (WJMSCs-apelin), and expression was verified by flow cytometry, Western blot, ELISA, and RT-PCR analysis. Type 2 diabetic rats were infused with either WJMSCs-apelin (2 × 10^6^ cells) or an equivalent dose of saline through the tail vein injection 7 days after STZ injection. The therapeutic effects of each infusion group were evaluated by monitoring plasma glucose levels and performing glucose tolerance tests (OGTTs), insulin tolerance tests (IPITTs), confocal microscopy, and immunocytochemical analysis for quantitating islet beta cells. Plasma inflammatory cytokines IL-6 and TNF-α and anti-inflammatory factors adiponectin were measured as well.

**Results:**

Type 2 diabetic rats infused with WJ-MSCs-apelin significantly decreased levels of blood glucose (from 26.03 ± 2.83 to 15.85 ± 2.13 mmol/L on 7 days *P* < 0.001, and to 9.41 ± 2.05 on 14 days, *P* < 0.001). Infusion of WJMSCs-apelin not only improved significantly insulin sensitivity and glucose disposal, but also promoted endogenous pancreatic ß cell proliferation (9.6-fold increase compared to the control group). Furthermore, infusion of the WJMSCs-apelin consistently increased insulin and C-peptide levels in the plasma, and the above effects persisted up to 42 days. The inflammatory cytokines IL-6 and TNF-α were significantly decreased, whereas anti-inflammatory factor adiponectin was significantly increased after WJ-MSC-apelin infusion.

**Conclusion:**

In this study, we report a novel approach to treat type 2 diabetic rats that combines apelin gene therapy with WJ-MSC cell therapy, which could provide a promising therapeutic option for management of type 2 diabetes clinically.

## Background

Type 2 diabetes mellitus (T2D) is a complex metabolic disease characterized by chronic hyperglycemia, insulin resistance, and pancreatic ß cell destruction [1–3]. While drug development has focused on restoring sensitivity over time and multiple treatment techniques have been developed, current therapeutic armamentarium is inadequate [4]. Development of novel therapeutic modalities is urgently required.

Apelin, a newly identified adipokine produced and secreted by adipocytes, regulates insulin sensitivity and glucose metabolism [5–7]. APLN gene encodes for a 77 amino acid pre-proprotein, which is processed to multiple active molecular forms including apelin-36, and apelin-13 being the most abundant and bioactive ones [8–10]. Apelin serves as an endogenous ligand of the ubiquitously expressed G protein-coupled receptor APJ [10, 11]. Apelin null (apelin−/−) mice exhibiting hyperinsulinemia and insulin resistance could be restored by the injection of apelin [6, 12]. Apelin stimulates glucose transport in an AMPK-dependent way in human adipose tissue explants and regulates glucose metabolism by promoting glucose absorption in the enterocytes [13]. In addition, apelin also acts directly on pancreatic islet and beta cells in rodents and human [14, 15]. These and other lines of evidence demonstrate that apelin may be a potentially viable candidate in the search for treatments for type 2 diabetes and the insulin resistance. While the therapeutic potential of the apelin is promising, the short half-life of apelin remains a limiting factor for their pharmacological use [16, 17]. Therefore, we applied viral vector gene delivery technologies to ensure a constant bioactive apelin expression and secretion.

Wharton’s jelly-derived mesenchymal stem cells (WJ-MSCs) are a primitive stromal cell population and have been isolated from a continuum from the sub-amnion to the perivascular region of the umbilical cord [18, 19]. WJ-MSCs retain a combination of the embryonic stem cell (ESC) and MSC markers in primary culture and early passages, thus retaining their multipotent stem cell characteristics [19, 20]. Recent studies have demonstrated that pancreatic endocrine precursor (PEP) cells can be generated from WJ-MSCs in the STZ rat model and human C-peptide-positive cells had been detected within the liver tissue 6 weeks following WJ-MSC transplantation [21, 22]. WJ-MSCs can regulate immunity by modulating the behavior of natural killer (NK) cells and T cell population and can secrete large numbers of cytokines, growth factors, and chemokines for improving microcircumstance [23]. Tilg et al. revealed a significant contribution to immunity induced by chronic inflammation that activates the infiltration of cells of the innate immune system in the pathogenesis of T2D and insulin resistance [24]. This suggests that WJ-MSCs might be more effective when combined with overexpression of apelin as mitigating ß-cell-specific immune response, having additive or synergistic effects on regeneration, immunoregulation, and restoration of insulin sensitivity.

To accomplish this, we engineered WJ-MSCs overexpressing apelin through a lentiviral vector and transfused T2D rats. We observed that apelin overexpression in WJ-MSCs reduced insulin resistance, promoted ß cell replication and function recovery, and improved microcircumstance of pancreatic islets.

## Methods

### Animals and induction of rat T2D model

Male Sprague-Dawley rats aged 7–8 weeks (160–180 g body weight) were purchased from the Department of Animal Resources of Chinese Academy of Military (Beijing, China). The laboratory animals were handled in accordance with Guidelines for the Care and Use of Laboratory Animals and the Animal Welfare Act in China and approved by both the Navy General Hospital Standing Committee and the Department of Animal Resources. The high-fat diet-fed, STZ-treated T2D rat model was established following published protocols [25]. Success of induction of T2D was verified by checking for blood glucose, glucose tolerance test, and insulin tolerance test, respectively. Diabetic rats were induced with a single intraperitoneal injection of STZ (60 mg/kg in 0.1 mol/L citrate buffer, pH 4.5), while the control group received saline of the same volume. Tail blood glucose was measured every 3 days during the experiments, and all animals with levels < 16 mmol/L were excluded from the diabetic group.

### Cell culture, flow cytometry analysis, and characterization of WJ-MSCs

WJ-MSCs were isolated and cultured as described previously [20]. The protocol was approved by the General Logistics Department of the PLA and the Navy General Hospital Ethical Review Board. Briefly, WJ-MSCs were harvested from passage 3, washed three times with PBS, and then added 0.1–10 μg/mL of conjugated antibody (Abcam, USA) followed by a 30-min incubation in dark at room temperature before flow cytometer analysis (Beckman, USA). In total, above 95% of cells expressed CD44, CD73, CD90, and CD105, while 2% or less of cells expressed CD45, CD34, and HLA-DR. Released cells were negative for pathogenic microorganisms, HBV, HCV, HIV, cytomegalovirus, syphilis, and ALT, and endotoxin levels were found within 40 IU/L and 0.5 EU/mL, respectively. The total cells were counted, and cell viability (≥ 85%) was determined by Trypan blue staining.

### Construction of apelin lentiviral expression plasmid and preparation of lentiviral particles

GV208-apelin (Genechem, China) was used for subclonning of apelin gene to the lentivector pReceiver-Lv203 (Genecopoeia, Rockville, MD, USA) with CMV promotor, enhanced green fluorescent protein (eGFP) and selection marker puromycin. Primers for amplifying the cDNA of the apelin gene (forward 5′-GCAGGCTTGGAAGGAGTTCGAACCATGAATCTGCGGCTCTGCGTGCAGGC-3′, reverse 5′-CATCGTCTTTGTAGTCCATTACTCTCAGAAAGGCATGGGTCCCTTATGGG-3′) were synthesized from Sangon (Shanghai, China). The pReceiver-Lv203-apelin clone was verified by DNA sequencing (GENEWIZ, China). Lentiviral particles with pReceiver-Lv203-apelin and pReceiver-Lv203 were produced through transfection of HEK293T packaging cells with 3rd generation plasmids system [26]. Lentiviral particles were harvested by ultrafiltration with 100 kDa spin column (Merck, Germany), dispensed in aliquots, and stored at − 80 °C until use. Transfection efficiency was examined by EGFP under a fluorescence microscope (Olympus, Japan), and viral titer was determined according to the equation: virus titer (pfu/mL) = cell number in each well × virus dilution factor × 10/volume of added virus fluid (mL).

### Transduction of WJ-MSCs with lentiviral particles and detection of apelin gene expression

Transduction of WJ-MSCs were performed in opti-MEM with lentiviral particles at MOI = 20 with polybrene (8 μg/mL). Transduction efficiency was determined as the percentage of GFP-positive cells in the total number of cells by flow cytometry analysis (FACS, BD Bioscience, USA) after 48 h. To detect the overexpression of the apelin in WJ-MSCs, Western blot analyses of cell lysates using anti-apelin monoclonal antibody were performed. To measure the secreted apelin, culture medium (CM) of WJ-MSCs, and WJ-MSCs transduced with pReceiver-Lv203-apelin, pReceiver-Lv203 lentiviral particles were collected after incubation for 48 h. The secreted apelin in the medium of WJ-MSC culture were measured by ELISA (Sigma, USA) according to the manufacturer’s protocol. For testing the proliferative effects of secreted apelin, WJ-MSCs were seeded in 96-well plates at 5 × 10^4^ cells/well and preconditioned in MEM-A medium for 2 days. Culture medium was then replaced with apelin-WJ-MSC CM, vector-WJ-MSC CM, or WJ-MSC CM. After 24-h incubation, 20 μl of 3-(4,5-dimethylthiazol-2y)-2,5-diphenyl-2H-tetrazolium bromide (MTT) was added to each well and incubated for 4 h at 37 °C. Supernatant was removed, and dimethyl sulphoxide (DMSO) was added to each well. Samples were shaken to dissolve the formazan, and absorbance was measured at 570 nm with a Quant microplate reader. All samples were analyzed as duplicates and samples with coefficient of variation (CV) values > 15% were excluded.

### Western blotting

The cells were washed with PBS and subsequently lysed using cell lysis buffer (Promega) with complete protease inhibitor mix (Roche). The cell lysate and control were run in SDS-PAGE gels (15%) respectively and transferred onto nitrocellulose membranes (Hybond-C Extra). Membranes were blocked with 5% milk in Tris-buffered saline plus Tween 20 (TBST) and exposed to antibodies against rabbit anti-apelin antibody (1:200, Abcam) and rabbit anti-GAPDH antibody (1:1000, Abcam). Blots were probed with horseradish peroxidase (HRP)-conjugated goat anti-rabbit IgG (H+L) secondary antibodies and visualized using a Bio-Rad Immun-Star Western C kit for signal detection.

### qRT-PCR of apelin expression

cDNA was synthesized using Maxime RT Premix kit (Intron biotechnology, Korea) with total RNAs isolated from WJ-MSCs transduced with lentiviral apelin or vector particles. Real-time quantitative PCR reactions were performed using SYBR green PCR Master Mix from Bio-Rad (Hercules, CA) using specific primers for human apelin (forward 5′-GTCTCCTCCATAGATTGGTCTGC-3′, reverse 5′- GGAATCATCCAAACTACAGCCAG-3). Triplicate reactions for the apelin and the endogenous controls (GAPDH) were performed separately on the same cDNA samples by using CFX Connect Real-Time System (Bio-Rad). The mean cycle threshold (C_t_) was used for the ΔΔC_t_ computations of the relative transcript abundance.

### WJ-MSC-apelin administration

Seven days after STZ injection, glucose tolerance tests and insulin tolerance tests were performed to validate the T2D rat model. Before WJ-MSC-apelin infusion, animals were randomized into three groups: (A) normal rats sham as control, (B) T2D rats that received only saline injection, and (C) T2D rats that received WJ-MSC-apelin injection. WJ-MSC-apelin infusion (2 × 10^6^ WJ-MSCs-apelin suspended in 0.5 mL physiological saline) or an equivalent dose of saline in T2D rats through the tail vein injection were performed. The investigators responsible for WJ-MSC-apelin and saline injections were blinded to the treatment groups.

### Determination of the effect of infused WJ-MSCs-apelin on hyperglycemia in T2D rats

Plasma glucose levels were monitored consecutively in alert, fasted rats using a glucometer ACCU-CHEK Advantage Meter (Roche Diagnostics GmbH, Mannheim, Germany). Six weeks after WJ-MSC-apelin infusion or saline infusion, glucose tolerance tests (OGTTs), insulin tolerance tests (IPITTs), and insulin release tests (IRTs) were performed. For glucose tolerance tests, rats were fasted for 6 h and administered intragastrically (2 mg glucose/g body weight). The blood glucose was measured from the tail tip using a One Touch Ultra 2 blood glucose meter (LifeScan, Inc.) at 0, 30, 60, 90, and 120 min postinjection. The area under the curve was calculated using standard methods [27]. For insulin tolerance tests, rats were injected intraperitoneally (2 g glucose/kg body weight) immediately followed by human insulin administration at a dose of 2 units/kg (Sigma).

### Confocal microscopy and immunocytochemical analysis

The pancreatic specimens of rats sacrificed at day 42 after treatment were fixed in 10% phosphate-buffered formalin and embedded in paraffin for histological analysis by hematoxylin-eosin and immunohistochemical staining. Cryostat sections (10 μm) were cut from frozen pancreatic tissue. For ß cell proliferation assays, sections were immunostained with anti-insulin and anti-Ki67 antibodies. All islets were imaged, and the total number of ß cells was counted by counting nuclei surrounded by cytoplasmic insulin immunostaining, and the number of proliferating ß cells was assessed by counting nuclei stained with Ki67 by cytoplasmic insulin immunostaining. Approximately 200 beta cells were counted for Ki67 analysis. The ß cell proliferation ratio was calculated by the number of Ki 67-positive cells divided by 200. For quantification of islet size and ß cell area, the whole areas of the all sections were imaged. The total pancreas area and insulin-positive area were selected using Adobe Photoshop. The total insulin-positive (ß cell) area and the average islet size (calculated from individual islet areas) from each image were calculated from these data for each rat. The average ß cell size was calculated with the total ß cell area (insulin^+^area) divided by the total ß cell number from all the imaged islets. Confocal images were obtained using a × 40 objective on a Leica Microsystems TCS SP laser scanning confocal microscope. Digitized confocal images were processed by Leica confocal soft-ware and Adobe Photoshop (version 6.0).

### Enzyme-linked immunosorbent assay (ELISA)

Blood samples were collected in three groups of rats at day 42 after WJ-MSC-apelin infusion, and serum and EDTA plasma were stored at − 20 °C. Concentrations of Apelin, C-peptide, and insulin in rat plasma were measured after WJ-MSC-apelin infusion. Plasma apelin concentrations of three groups of rats were quantified with an ELISA kit (Phoenix Pharmaceuticals, Belmont, CA, USA) following the manufacturer’s instructions. The concentrations of interleukin-6 (IL-6), tumor necrosis factor-α (TNF-α), and adiponectin (APN) were determined with ELISA kits (R&D Systems, Minneapolis, MN, USA). All samples were analyzed with duplicates and samples with coefficient of variation (CV) values > 15% were excluded. Blood biochemical analyses and laboratory assays including hematologic, tumor, and immune indexes were conducted by routine clinical lab methods at 6 weeks after treatments.

### Statistics

An unpaired Student’s *t* test or one-way ANOVA test was used to determine statistical significance. Post hoc analysis was performed using the Bonferroni method. Values of *P* < 0.05 were considered statistically significant. All experiments in vitro (real-time RT-PCR, Western blot, and immunofluorescence assay) were representative of three independent experiments. Data were expressed as mean ± SE. Dunnett *t* test or two-way ANOVA were used for statistics analysis as appropriate. Comparisons between groups were made using Dunnett *t* test or two-way ANOVA. *P* value < 0.05 was considered significant.

## Results

### Morphology and immunophenotypic characterization of WJ-MSCs

**Fig. 1:**
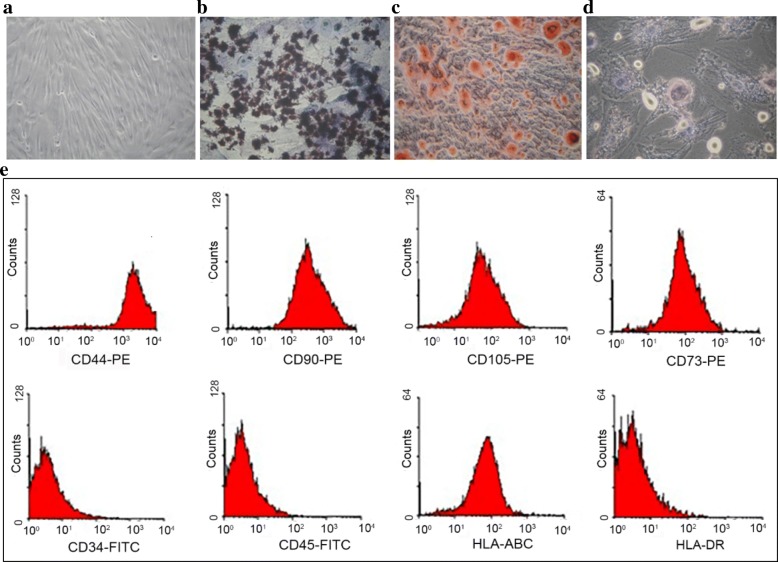
Morphology and multilineage differentiation capacity of WJ-MSCs. **a** WJ-MSCs showed a homogeneous spindle-shaped morphology. **b** Osteogenesis was examined by von Kossa staining for mineral nodule deposition. **c** Chondrogenesis was assessed by Safranin O staining for proteoglycan deposition. **d** Adipogenesis was observed by the presence of lipid vesicles and confirmed by oil red O staining. **e** Immunophenotype of WJ-MSCs by flow cytometric analysis. Representative histograms are demonstrated. WJ-MSCs were positive for CD44, CD90, CD105, CD73, and HLA-ABC and negative for CD34, CD45, and HLA-DR

WJ-MSCs isolated from human Wharton’s jelly with phase-contrast microscopy display a uniform spindle-shaped morphology like fibroblastoid cells (Fig. 1a). In vitro differentiation analysis confirmed that WJ-MSCs exhibited the capacity to differentiate into various cell types, such as osteoblasts, chondrocytes, and adipocytes (Fig. 1b–d). For further characterization of WJ-MSCs, a panel of surface markers were tested using flow cytometric analysis. WJ-MSCs were negative for CD34, CD45 (both as hematopoietic markers), and HLA-DR (human leukocyte antigen class II), whereas they were positive for CD44, CD73, CD90, CD105, and HLA-ABC (Fig. 1e).

### Apelin expression in transduced WJ-MSCs

**Fig. 2:**
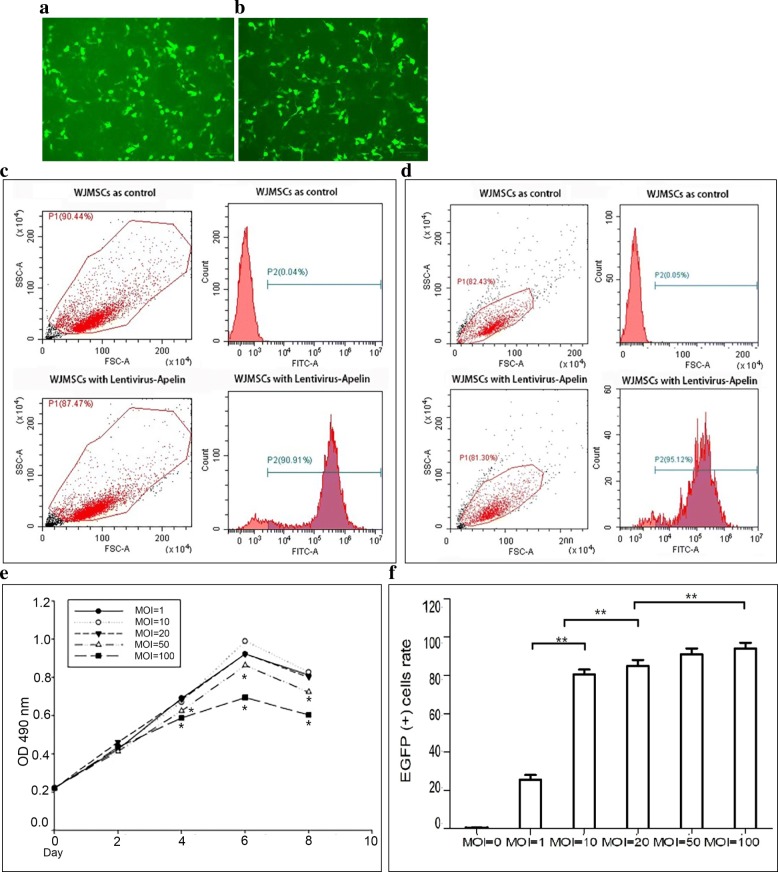
Transduction of WJ-MSCs with lentiviral vector particles for apelin expression. **a**, **b** Expressing of EGFP in transduced WJ-MSCs with MOI of 20 and 50 under fluorescence microscopy. **c** Analysis of EGFP fluorescence by flow cytometry at 48 h after transduction. The upper panel represents the nontransduced WJ-MSCs. **d** Apelin expression was detected by flow cytometry analysis using monoclonal anti-apelin antibody at 48 h after transduction. The upper channel represents the nontransduced WJ-MSCs. **e** Proliferation rate of the EGFP-positive transduced WJ-MSCs is significantly different among the cells with different MOI. **f** Cell growth of the transduced WJ-MSCs with different MOI was significantly different via MTT assay at OD 490 nm

WJ-MSCs were transduced with the lentiviral vectors pReceiver-Lv203 expressing EGFP as a marker. Transduced cells were examined for EGFP using a fluorescence microscope (Fig. 2a and b). Flow cytometry analysis of EGFP and apelin was performed with the cells at 48 h after transduction, and EGFP-positive cells ranged from 90 to 95% (Fig. 2c, d). FACS analysis showed that EGFP-positive cells were over 90% when multiplicities of infection (MOI) was 20, and transfection efficiency was not significantly raised between MOI 20 and 50 (*P* > 0.05) and no increase to raise MOI to 50 or 100 (*P* > 0.05; Fig. 2e). Meanwhile, MTT assay showed that the proliferation of transfected cells was affected when MOI was over 50 (*P* < 0.05; Fig. 2f). Consequently, the optimal MOI for transduction protocol was 20.

**Fig. 3:**
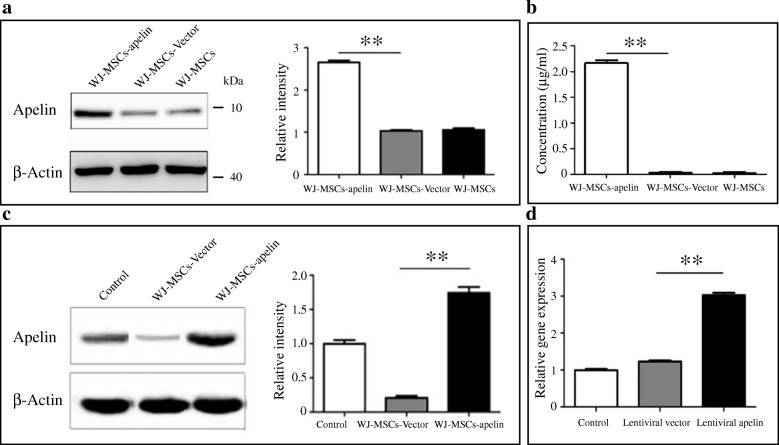
Detection of overexpression of apelin in transduced WJ-MSCs. **a** Western blot analysis shows strong apelin band in the WJ-MSCs transduced with apelin expressing lentiviral particles compare to the vector lentiviral particles or nontransduced WJ-MSCs. The intracellular GAPDH protein are used as a loading controls; **b** ELISA analysis of the apelin level in the cell culture media of apelin-transduced WJ-MSCs compared to the media of the nontransduced WJ-MSCs. **c**, **d** Quantitative analysis of apelin expression level of mRNA via RT-PCR of apelin in the apelin-transduced cells, vector-transduced cells, and the nontransduced WJ-SCs. The values are mean ± SD from four individual experiments. **P < 0.01

To further demonstrate the overexpression of apelin, Western blot analyses of cell lysates from apelin-transduced WJ-MSCs and lentiviral vector-transduced WJ-MSCs were carried out. As expected, we observed more than 2.6-fold change of the apelin in cell lysate from apelin-transduced WJ-MSCs compare to the vector-transduced cells (Fig. 3a). The concentration of secreted apelin in the culture medium of WJ-MSCs transduced with apelin was significantly higher than the one in the WJ-MSCs transduced with vector by ELISA analyses (Fig. 3b). Furthermore, quantitative RT-PCR and cDNA densitometry analysis confirmed that apelin mRNA was found expressed much higher in apelin-transduced WJ-MSCs compare to vector-transduced WJ-MSCs (Fig. 3c, d).

**Fig. 4:**
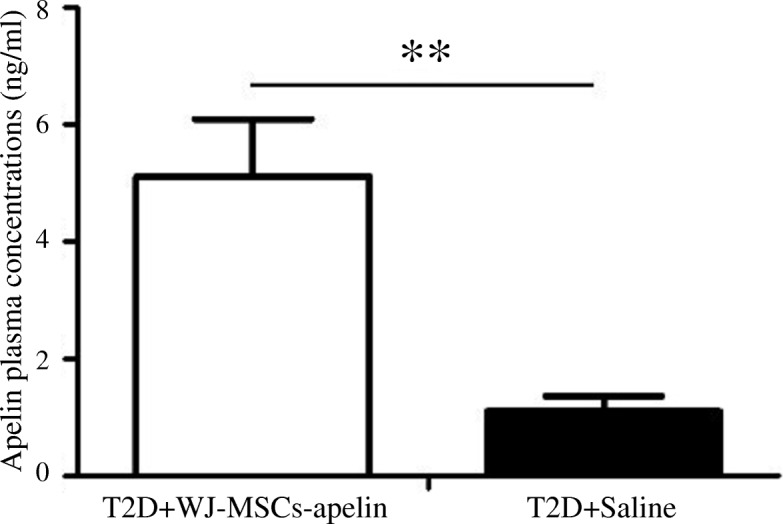
In vivo detection of plasma apelin levels in saline-infused and apelin-transduced WJ-MSCs infused T2D rats after 42 days of treatment. *n* = 10 rats per group. Apelin levels are reported as mean and 95% CI (confidence interval). T2D+saline: type 2 diabetic rat with saline infusion; T2D+WJMSCs-apelin: type 2 diabetic rat with Wharton’s jelly mesenchymal stem cell-apelin infusion

We examined the apelin plasma level in rats infused with apelin-transduced WJ-MSCs and the saline infusion group. Apelin concentrations were increased 4.65-fold in the plasma of rats infused with apelin-transduced WJ-MSCs at 42 days compare to the saline infusion group (5.12 ± 0.98 vs. 1.10 ± 0.26 ng/mL) (Fig. 4). Sustained apelin expression was observed in the rats infused with apelin-transduced WJ-MSCs for up to 42 days.

### Overexpression of apelin in WJ-MSCs significantly improved hyperglycemia and survival in T2D rats

**Fig. 5:**
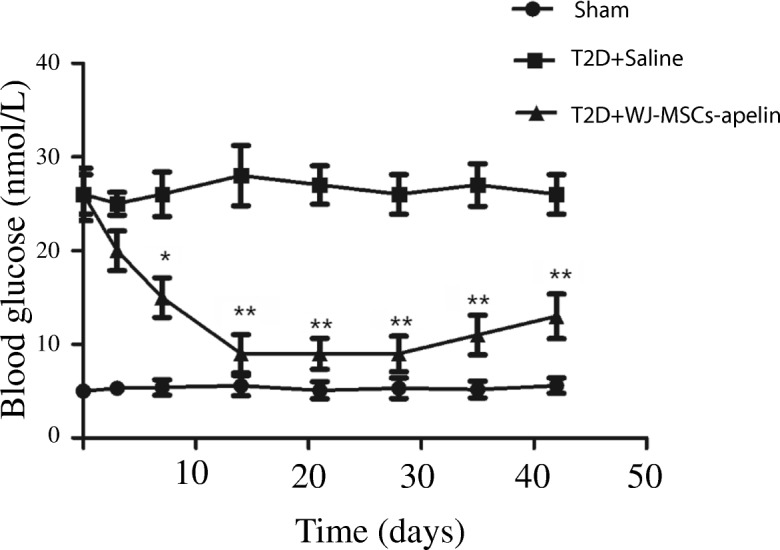
WJ-MSC-apelin infusion significantly improved hyperglycemia in T2D rats. Blood glucose level was determined consecutively up to 42 days in alert, fasted rats using a glucometer ACCU-CHEK Advantage Meter. The levels of blood glucose were improved gradually but significantly after 7 days in WJ-MSC-apelin infusion, but not in saline infusion T2D rats. *n* = 10 rats per group. *P < 0.05 and **P < 0.01. T2D+saline: type 2 diabetic rat with saline infusion; T2D+WJMSCs-apelin: type 2 diabetic rat with Wharton’s jelly mesenchymal stem cell-apelin infusion

No signs of an immune response triggered by the allogeneic WJ-MSC-apelin infusion, ectopic tissue formation, or increased levels of tumor-associated antigens were observed in the study (Tables 1 and 2). During the experimental period, the blood glucose levels of the rats were constantly monitored throughout the experiments and the saline-treated T2D rats showed persistent hyperglycemia, combined with gradual decrease in body weight and higher mortality, as compared to those T2D rats with the WJ-MSC-apelin infusion. The levels of blood glucose were improved gradually but significantly after 7 days of WJ-MSC-apelin infusion (Fig. 5). The average fasting blood glucose level decreased from initial 26.03 ± 2.83 to 15.85 ± 2.13 mmol/L by day 7 (*P* < 0.001) and further reduced to 9.41 ± 2.05 by day 14 (*P* < 0.001). The effect of lowering blood glucose continued for 42 days (Fig. 5). These results suggested that WJ-MSC-apelin infusion contributed to amelioration of hyperglycemia in T2D rats.

**Table 1:** Comparison of hematological parameters between rats infused with T2D+saline and T2D+WJMSCs-apelin after 42 days

Item	Group					
	T2D+WJMSCs-apelin (B)	T2D+saline (D)	Sham (N)	*P* value (B&D)	*P* value (B&N)	*P* value (D&N)
High-density lipoprotein	1.06 ± 0.11	0.97 ± 0.36	1.03 ± 0.09	0.43	0.46	0.64
Low-density lipoprotein	0.38 ± 0.07	0.38 ± 0.27	0.41 ± 0.09	0.97	0.42	0.73
Serum total protein	63.36 ± 2.73	76.59 ± 25.83	66.01 ± 4.20	0.09	0.09	0.22
Alanine aminotransferase	53.50 ± 10.68	61.59 ± 50.27	59.66 ± 14.66	0.59	0.28	0.91
Total cholesterol	2.02 ± 0.18	2.43 ± 1.07	1.77 ± 0.39	0.21	0.55	0.08
Triglyceride	2.32 ± 1.25	5.31 ± 7.60	0.98 ± 0.56	0.09	0.005	0.09
Serum albumin	29.11 ± 1.32	32.13 ± 5.73	33.95 ± 2.65	0.09	< 0.0001	0.37
Urea nitrogen	5.78 ± 0.68	10.63 ± 11.91	6.63 ± 0.70	0.17	0.01	0.30
Creatinine	44.65 ± 3.59	66.84 ± 41.86	43.34 ± 2.65	0.08	0.35	0.09
Uric acid	60.08 ± 44.16	49.17 ± 37.49	72.00 ± 34.36	0.52	0.50	0.16
TNF-α (pg/mL)	14.3 ± 3.14	51.2 ± 9	8.22 ± 2.11	0.007	0.003	0.004
IL-6 (pg/mL)	50.21 ± 3.7	147.6 ± 7.13	42.03 ± 4.6	0.0002	0.011	< 0.0001
APN (μg/mL)	9.36 ± 2.21	6.46 ± 5.10	11.68 ± 2.17	0.11	0.012	0.027

Plus–minus values are means ± SE. *P* values of between-group comparisons were determined by Student’s *T* test or nonparametric Mann–Whitney *U* tests. *P* values of within-group comparisons were determined by ANOVA with 95% CIs. *P* > 0.05 not statistically significant. *B*, type 2 diabetic rats infused with WJMSCs-apelin; *D*, type 2 diabetic rats infused with saline; *N*, sham; *T2D+saline*, type 2 diabetic rat with saline infusion; *T2D+WJMSCs-apelin*, type 2 diabetic rat with Wharton’s jelly mesenchymal stem cell-apelin infusion. *B&D*, comparison between T2D-WJMSCs-apelin and T2D-saline; *B&N*, comparison between T2D-WJMSCs-apelin and sham; *D&N*, comparison between T2D-saline and sham

**Table 2:** Comparison of immunological parameters between rats infused with T2D+saline and T2D+WJMSCs-apelin before and after 42 days

Parameter	T2D+saline		T2D+WJMSCs-apelin		Sham
	Prior treatment	42 days after treatment	Prior treatment	42 days after treatment	
IgG(g/L)	11.3 ± 0.8	10.6 ± 0.9	11.0 ± 0.7	10.9 ± 0.6	10.8 ± 0.4
IgM(g/L)	1.13 ± 0.08	1.14 ± 0.11	1.10 ± 0.12	1.15 ± 0.14	1.08 ± 0.07
CD3(%)	65.8 ± 3.1	64.6 ± 2.2	66.1 ± 3.4	67.2 ± 4.7	66.3 ± 3.9
CD4(%)	39.2 ± 1.3	38.1 ± 1.7	38.9 ± 1.1	38.2 ± 1.6	39.3 ± 1.5
CD8(%)	22.9 ± 0.9	23.4 ± 1.2	22.4 ± 0.8	22.1 ± 1.4	22.7 ± 0.7

No significant differences were shown in comparison of baseline with values measured after 42 days of rats with WJ-MSC-apelin or saline infusion. *T2D+saline*, type 2 diabetic rat with saline infusion; *T2D+WJMSCs-apelin*, type 2 diabetic rat with Wharton’s jelly mesenchymal stem cell-apelin infusion

### Overexpression of apelin in WJ-MSCs improved insulin sensitivity in T2D rats

**Fig. 6:**
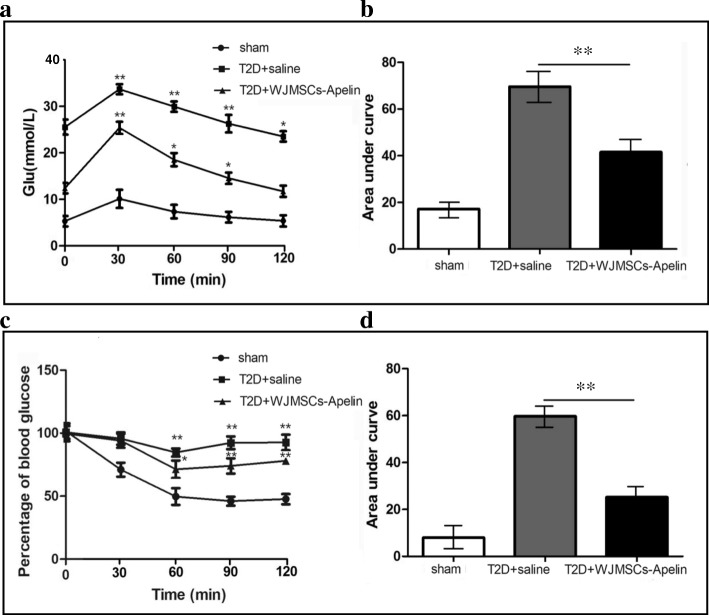
WJMSC-apelin infusion improved insulin sensitivity in T2D rats. **a** Individual oral glucose tolerance was assessed by OGTTs 6 weeks after MSC-apelin infusion. T2D rats with WJMSC-apelin infusion (*n* = 5) have lower fasting blood glucose and an improved glucose tolerance compared with saline infusion group. **b** Area under curve (AUC) for the glucose tolerance test is calculated using standard methods. **c** Insulin tolerance was evaluated by IPITTs in T2D rats with WJMSC-apelin or saline infusion (each group, *n* = 5). There was a severe insulin resistance in T2D-saline rats, but no sign of insulin resistance in WJMSC-apelin infusion T2D rats. **d** AUC for insulin tolerance is calculated using standard methods. T2D+saline: type 2 diabetic rat with saline infusion; T2D+WJMSC-apelin: type 2 diabetic rat with Wharton’s jelly mesenchymal stem cell-apelin infusion

To determine the effects of WJ-MSC-apelin fusion on insulin sensitivity, a glucose tolerance test was performed in three groups. Rats were fasted for 6 h before glucose injection. The apelin-transduced WJ-MSC rats have a lower fasting glucose level and improved glucose tolerance compared to saline-injected T2D rats (Fig. 6a, b). Furthermore, we performed insulin tolerance tests (IPITTs) and area under the curve at 6 weeks on rats after WJ-MSC-apelin infusion. T2D rats that received WJ-MSC-apelin infusion showed better insulin sensitivity and faster glucose disposal represented by significantly lower blood glucoses compared with T2D rats with saline infusion at various times and area under the curve after glucose administrated intragastrically (Fig. 6c, d). These data suggest that a single WJ-MSC-apelin fusion improved insulin sensitivity and provided protective effects that persisted long after the treatment had ended.

### Overexpression of apelin in WJ-MSCs promotes endogenous pancreatic ß cell proliferation

**Fig. 7:**
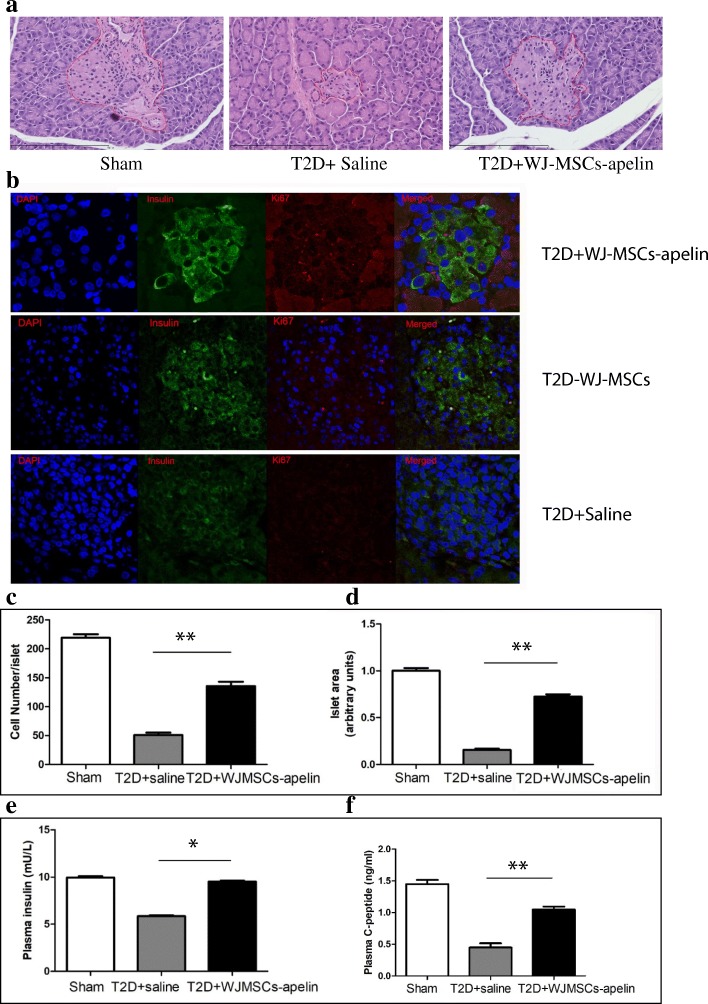
WJMSC-apelin infusion leads to a pancreatic ß cell proliferation in T2D rats. **a** HE staining showed that the size of islets is normal and the islets are intact and full in sham rats. In T2D+saline rats, pathological display showed that pancreatic islets appeared moderate to severe atrophy, and the islet structure shrank. The islet cells were greatly reduced and distributed evenly. In WJMSC-apelin infusion rats, mild atrophy of whole islets was observed and the islet structure is not complete. Some islet cells distributed evenly, part of uneven density. **b** Representative images showing the colocalization of Ki67+ with insulin+ cell in the pancreatic islets of the WJMSC-apelin infusion T2D rats and saline infusion T2D rats. Nuclei were stained by DAPI (blue). Insulin (green) and Ki-67+ (red). WJMSC-apelin infusion strongly stimulates ß cell replication compared to the saline infusion control in T2D rats (each group *n* = 5). **c**, **d** Quantification of the insulin+ cells and the islet area from pancreatic islets (10 sections) by counting the numbers of positive cells per 100 nuclei per rat 42 days after WJMSC-apelin or saline infusion. Magnification × 40; bars indicate 95% confidence intervals (CIs). *P < 0.05, **P < 0.01. **e**, **f** ELISA analysis of insulin and C-peptide level in individual fasted rats 42 days after WJMSC-apelin or saline infusion. *n* = 10 rats per group. *P < 0.05 and **P < 0.01

The pancreatic islets appear moderate to severe atrophy, and the islet structure shrinks, and the islet cells were greatly reduced and distributed evenly in T2D+saline group rats by HE staining while the size of islets is normal and the islets are intact and full in sham rats (Fig. 7a). However, in T2D rats with a single WJ-MSC-apelin fusion, pancreatic islets showed mild atrophy with incomplete islet structure and some islet cells distributed evenly, part of uneven density (Fig. 7a).

To identify whether overexpression of apelin can promote endogenous pancreatic ß cell proliferation and replication, we performed insulin/Ki67 double immunofluorescence examination. The ß cell proliferation rate in T2D rats with a single WJ-MSC-apelin fusion is 2.6%, 9.6-fold higher than the rate in control T2D rats (0.27%, Fig. 7b). The higher ß cell proliferation rate in T2D rats with WJ-MSC-apelin fusion leads to a significant expansion of ß cell numbers and total pancreatic ß cell mass. This increase resulted in more ß cells, which in turn enlarges the islet size (Fig. 7c, d) and consequently raised the plasma content of insulin and C-peptide in T2D rats with WJ-MSC-apelin fusion (Fig. 7e, f).

### Overexpression of apelin in WJ-MSCs attenuated plasma levels of inflammation factors in T2D rats

Inflammatory cytokines IL-6 and TNF-α have been implicated in insulin resistance. Plasma levels of TNF-α and IL-6 detected by ELISA were significantly higher (*P* < 0.01) in the T2D rats, as compared to those in the sham group (Table 1). However, plasma levels of TNF-α and IL-6 were significantly lower in T2D rats with WJ-MSC-apelin fusion up to the 42nd day (Table 1). Moreover, plasma level of adiponectin, a fatty cytokine secreted by adipocytes mediating insulin sensitizing, was significantly increased (*P* < 0.01) as well compared to the level in the rats with saline injection (Table 1).

## Discussion

Apelin, a endogenous regulatory peptide hormone, and its receptor APJ are widely expressed in the body and play cytoprotective roles in multiple physiological processes [28, 29]. Recent studies demonstrated that apelin is involved in energy metabolism and the pathophysiology of obesity [7]. Direct injection of apelin-13 or its modified analogues improves glucose handling and insulin sensitivity [12]. Native apelin undergoes extensive enzymatic degradation and is cleared rapidly from the circulation with a half-life of < 5 min in humans [17, 30], which limits its therapeutic benefit. Bioactive analogues did extend the plasma half-life with greater duration of antihyperglycemic actions; however, daily treatment was required in rodents [31].

As per our knowledge, we are the first one to report that WJ-MSCs could potentially be used as an apelin delivery system to merge the useful biological functions of apelin with the beneficial therapeutic effects of WJ-MSCs. We found that infusion of apelin-transduced WJ-MSCs to T2D rats not only improved significantly insulin sensitivity and glucose disposal, but also promoted endogenous pancreatic ß cell proliferation, as well as raised plasma content of insulin and C-peptide. Furthermore, the above effects persist up to 42 days in the rats after a single infusion with the apelin-transduced WJ-MSCs. Given the fact that apelin has therapeutic abilities in the treatment of multiple disease including metabolic disorders [28], we believe we introduced a potential novel cell therapy for treating T2 diabetes mellitus clinically.

Wharton’s jelly-derived mesenchymal stem cells (WJ-MSCs) retain a combination of most of their embryonic stem cell (ESC) and MSC markers in primary culture and early passages, thus retaining their multipotent stem cell characteristics [18–20]. The human umbilical cord-derived mesenchymal stem cells (HUCMSCs) can differentiate into insulin-producing cells in vitro [21, 22]. Using a portal vein injection, HUCMSC-differentiated islet cells can alleviate hyperglycemia in diabetic mice [21] and rats [22]. In patients with type 2 diabetes mellitus who received WJ-MSCs, all parameters improved, HbA1c level gradually decreased, and progressive increase of C-peptide and C-peptide/glucose ratio was observed [32].

In our experiments, we transduced WJ-MSCs with apelin using lentiviral expression. We only observed moderate overexpression of apelin in the cells that could be due to its secretion since there is a signal peptide present in the expression construct. The moderate expression of apelin also could be contributed by the CMV promoter inefficiently driven in WJ-MSCs [33, 34] and most possibly due to its short half-life [35]. Injection of apelin decreased glycemia in normal-chow diet (ND)-fed as well as fasted mice mainly due to increased glucose uptake in targeted tissues like skeletal muscle, through an AMP-activated protein kinase (AMPK)-dependent pathway [6, 12]. In addition, apelin also increased phosphor-Akt in muscle manner both ex vivo [12] and in vivo [6]. Moreover, apelin could also regulate glucose metabolism by promoting glucose absorption in the enterocytes and then by increasing portal blood glucose and insulin secretion [36]. This agrees with the fact that apelin was shown to increase GLP-1 secretion [36]. We hypothesized that the overexpression of apelin in WJ-MSCs may present additive or synergistic beneficial effects on glucose uptake, improving insulin sensitivity in T2D rats.

To identify whether overexpression of apelin in WJ-MSCs can promote endogenous pancreatic ß cell proliferation and replication, we performed insulin/Ki67 double immuno-fluorescence staining. The increase in ß cell proliferation was observed in all islets examined. The high ß cell proliferation rate leads to a significant expansion of ß cell numbers and total pancreatic ß cell mass after 42 days of treatment (Fig. 7). Plasma content of insulin and C-peptide were higher in T2D rats infused with apelin-transduced WJ-MSCs.

The mechanism of apelin-promoted ß cell proliferation has yet to be determined. The fact that apelin was able to increase Akt phosphorylation in muscle as well as in adipocytes [6, 12, 37], it is perceivable that apelin could activate PI3K/Akt pathway to promote pancreatic ß cell proliferation [38]. Here, we confirmed that WJ-MSCs-apelin was able to enhance mobilization, survival, and proliferation of endogenous pancreatic ß cell in the injured islets of the pancreas, suggesting a novel additive or synergistic mechanism for possible explanation how WJ-MSCs-apelin might repair the pancreas islets and improve pancreatic ß cell function in T2D rats.

Multiple analyses found no major safety concerns regarding mesenchymal stem cell (MSC) administration to treat patients with T2DM and other diseases, and no association between MSC treatments and tumor formation was found [39]. Allogeneic WJ-MSC transplantation in patients with T2DM was also proved to be safe [32, 40]. We transduced WJ-MSCs with a lentiviral vector for genomic integration of APLN gene resulting to the risk of genomic disruption that poses a challenge for WJ-MSC-based transplantation therapy. We found no difference of cell proliferation between the apelin-transduced WJ-MSCs and nontransduced WJ-MSCs before injection to T2D rats (data not shown). WJ-MSC-apelin’s infusion is safe and induced neither acute nor persistent immune or biochemical abnormalities in T2D rats (Table 1).

At present, many studies have implicated cytokine-mediated inflammation in the pathogenesis of insulin resistant and T2DM [41]. Chronic inflammation has been recognized as a critical inducer in the development of insulin resistance and T2D [42, 43]. Several inflammatory cytokines, such as tumor necrosis factor-α (TNF-α) and interleukin-6 (IL-6), have been implicated in the causation of insulin resistance and appear to confer an increased risk of microvascular complications in type 2 diabetes mellitus [44]. Consistently with other studies, the TNF-α and IL-6 levels were significantly higher in the T2D rats, as compared to those in the sham group in our experiments. However, TNF-α and IL-6 levels were significantly decreased in the T2D rats infused with apelin-transduced WJ-MSCs. Adiponectin (APN) is a fatty cytokine secreted by adipocytes, which is currently regarded as biological effect: (1) insulin sensitizing (insulin metabolism adjusting) and (2) anti-inflammatory. A large number of epidemiological studies have found that most of patients with T2DM have hypoadiponectinemia [45]. The serum apelin level was significantly lower in the T2D rats, but significantly increased in T2D rats after infusion of apelin-transduced WJ-MSCs (*P* < 0.01) that reduced the risk of hypoadiponectinemia. Our experiments in vivo suggested that apelin-transduced WJ-MSCs might play an anti-inflammatory role in T2D rats, which improved the sensitivity of insulin.

## Conclusions

In conclusion, the present study has shown a new approach that combines apelin gene therapy with WJ-MSC cell therapy to treat type 2 diabetic rats effectively. We found that infusion of apelin-transduced WJ-MSCs to T2D rats improved significantly insulin sensitivity and glucose disposal, promoted endogenous pancreatic ß cell proliferation, and increased plasma content of insulin and C-peptide.

## Abbreviations

ALT: Alanine transaminase
BM-MSCs: Bone marrow-derived mesenchymal stem cell
CM: Culture medium
CV: Coefficient of variation
EGFP: Enhanced green fluorescent protein
ELISA: Enzyme-linked immunosorbent assay
FACS: Flow cytometry analysis
HBV: Hepatitis B virus
HCV: Hepatitis C virus
HE: Hematoxylin and eosin
HIV: Human immunodeficiency virus
IL-6: Interleukin-6
IPITTs: Insulin tolerance tests
OGTTs: Glucose tolerance tests
STZ: Streptozotocin
T2D: Type 2 diabetes
WJ-MSCs: Wharton’ jelly mesenchymal stem cells
