# Correction to: Overexpression of apelin in Wharton’ jelly mesenchymal stem cell reverses insulin resistance and promotes pancreatic β cell proliferation in type 2 diabetic rats

**DOI:** 10.1186/s13287-018-1116-6

**Published:** 2019-01-08

**Authors:** Lian Ru Gao, Ning Kun Zhang, Yan Zhang, Yu Chen, Li Wang, Ying Zhu, Hai Hong Tang

**Affiliations:** 1 Center of Cardiology, The Sixth Medical Center of P.L.A. General Hospital (Former Navy General Hospital), NO.6 Fucheng Road, Beijing, Haidian District 100048 People’s Republic of China; 2 Department of Internal Medicine, The 413th Hospital of P.L.A., 98 Wenhua Road, Zhoushan, Zhejiang 316000 People’s Republic of China

**Correction to: Stem Cell Res Ther**

**https://doi.org/10.1186/s13287-018-1084-x**

The original article [1] contained errors in the presentation of Figure 7. These have now been corrected.
